# Aerosol Sampling in a Hospital Emergency Room Setting: A Complementary Surveillance Method for the Detection of Respiratory Viruses

**DOI:** 10.3389/fpubh.2018.00174

**Published:** 2018-06-14

**Authors:** Jessica Y. Choi, Juliana Zemke, Sarah E. Philo, Emily S. Bailey, Myagmarsukh Yondon, Gregory C. Gray

**Affiliations:** ^1^Duke Global Health Institute, Duke University, Durham, NC, United States; ^2^Division of Infectious Diseases, Duke University School of Medicine, Durham, NC, United States; ^3^Global Health Research Center, Duke-Kunshan University, Kunshan, China; ^4^Emerging Infectious Diseases Program, Duke-NUS Medical School, Singapore, Singapore

**Keywords:** infectious aerosols, bioaerosol sampling, emergency service, hospital, epidemiology, respiratory viruses

## Abstract

This study aimed to evaluate environmental air sampling as an alternative form of active surveillance for respiratory pathogens in clinical settings. Samples were collected from three locations in the Emergency Department at Duke University Hospital Systems from October 2017 to March 2018. Of the 44 samples collected, 12 were positive for known respiratory pathogens including influenza A, influenza D, and adenovirus. Results suggest bioaerosol sampling may serve as a complement to active surveillance in clinical settings. Additionally, since respiratory viruses were detected in aerosol samples, our results suggest that hospital infection control measures, including the use of N95 respirators, could be used to limit the spread of infectious viruses in the air.

## Introduction

Emergency Departments (ED) are often at the frontline of clinical care for many who are seriously ill, unable to be seen acutely by a primary care provider, or are uninsured (1). Particularly during respiratory virus and seasonal influenza outbreaks, EDs are tasked with the evaluation and treatment of sick individuals. As there is increasing evidence to suggest that respiratory viruses may be transmitted in air (2–4), novel surveillance targeting bioaerosols has been suggested as a non-invasive clinical sampling technique (5).

In this pilot study, we studied bioaerosol samples collected in the ED for molecular evidence of respiratory viruses. Our overall goal was to determine if environmental air sampling was a viable alternate method for respiratory virus surveillance in clinical settings.

## Materials and methods

### Ethics approval and study location

This study was granted exemption from review status by the Institutional Review Board at Duke University on the grounds that the research did not directly involve contact with human subjects. Permission from the clinical supervisor at Duke Emergency Department was obtained and collaboration with the on-duty care providers was ensured in order to conduct this study at Duke University Hospital Emergency Department.

### Bioaerosol sampling

Bioaerosol sampling at Duke Hospital ED was conducted by study personnel once a week from October 4th 2017 to March 1st 2018, excluding a 6-week holiday period from the beginning of December 2017 to mid-January 2018. In total, 15 separate sampling periods yielded 44 aerosol samples to be tested for the panel of viruses.

Environmental air was circulated through National Institute of Occupational Safety and Health (NIOSH) BC 251 Personal Aerosol Samplers by AirCheck XR5000 Sample Pumps (Cat. # 210-5000, SKC, Inc., Eighty-Four, PA). Each NIOSH Sampler featured two stages of collection for pathogens greater than 4 and 1–4 μm in size, and a polytetrafluoroethylene (PTFE) back-up filter (0.03 μm pore, 37 mm) to capture pathogens less than 1 μm, producing three specimens for each sampler during each run. Pathogens filtered greater than 4 μm and 1–4 μm had the potential for remaining viable after capture.

The samplers were placed 1.5 m above the ground, approximately at eye-level for seated visitors, at three locations within the ED. Two samplers were placed in the Duke ED waiting room, one at each of the North and South wings of the ED waiting room seating area. The third sampler was set-up in one of the available triage rooms (1, 2, or 3) used for the rapid assessment of patient symptoms. The sampling pumps were calibrated to a flow rate of 3.5 L/min and ran for approximately 100–150 min during each sampling session. At the end of the sampling session, the location of sampling was recorded with the data, time, sampler number, pump number, and run time.

### Sample processing

Upon completion of the sampling period, all bioaerosol samplers were transported back to the Duke One Health Research Laboratory (located in an adjacent building in the medical complex). The filter and catchment containers were processed as previously described (5). Briefly, filter and catchment containers were removed from the samplers, rinsed with a sterile virus collection medium (PBS with 0.5% BSA) and aliquoted into 2.0 mL cryovials for storage. Samples were stored at −80°C until further molecular work was performed.

### Molecular assays

We focused this pilot study surveillance upon four groups of prevalent respiratory viruses, human and animal. Published real-time polymerase chain reaction (qPCR) and real-time reverse transcription polymerase chain reaction (qRT-PCR) assays for influenza A/B/C/D viruses, human adenoviruses, human enteroviruses, and human coronavirus were used with DNA or cDNA positive controls and nuclease-free water as negative controls (Table S1).

Extraction of viral RNA from the stored samples was completed using the QIAmp Viral RNA Mini Kit (QIAGEN, Inc., Valencia, CA) and tested with qRT-PCR assays using Superscript® III Platinum One-Step qRT-PCR System with Platinum® Taq DNA Polymerase (Thermo Fisher Scientific, Inc., Waltham, MA) for detection of Influenza A (6, 7), influenza B (6, 8), influenza C (9), influenza D (6), human coronavirus (10, 11), and human enterovirus (10). Pan-species coronaviruses (10) were detected with gel-based RT-PCR assays using Superscript® III Platinum One-Step RT-PCR System with Platinum® Taq DNA Polymerase (Thermo Fisher Scientific, Inc., Waltham, MA).

For the analysis of specimens for adenovirus (11), viral DNA was extracted using the QIAamp DNA Blood Mini Kit (QIAGEN, Inc. Valencia, CA) and then examined by a real time PCR (qPCR) assay (12) using the Sso Advanced Universal Probes Supermix (Bio-Rad, Hercules, CA). Positive specimens were then confirmed using previously described two step molecular assays focusing on the hexon gene (13). Extracted viral DNA was also tested with for detection of pan-species adenovirus (14) with Platinum® Taq DNA Polymerase Kit (Thermo Fisher Scientific Inc., Waltham, MA). Amplified product from both the hexon assay and the pan-species assay was submitted to Eton Bioscience (Eton Bioscience, Inc., Raleigh, NC, USA) for sequencing. Using BioEdit 7.1.9 (Ibis Biosciences, Carlsband, CA, USA) sequences were aligned, edited, and then compared to the National Center for Biotechnology Information (NCBI) sequence database using the BLAST application.

## Results

From October 2017 to March 2018, a total of 44 bioaerosol samples were collected from three sites in the emergency room of Duke hospital. Overall, 12 (27%) of the 44 samples indicated evidence of at least one respiratory pathogen over a period of 8 different sample collection days (Table 1). One sample was positive for influenza A virus (2%), one was positive for influenza D virus, and 10 (23%) samples were positive for adenovirus. Five of the 10 adenovirus positive specimens were successfully sequenced using partial hexon sequencing and found to be either human adenovirus type 1, 7, or 21, as reported in Table 2. Adenoviruses were detected most frequently in the North wing of the ED waiting room seating area, with 5 (11%) positive samples detected in that area.

**Table 1:** Molecular results for three sites in the emergency department at Duke University Hospital, October 2017 to February 2018.

**Sample ID**	**Date**	**Site**	**FluA**	**FluB**	**FluC**	**FluD**	**AdV**	**PanAdV**	**CoV**	**PanCoV**
DM-BS013	10/4/2017	North	–	–	–	–	–	–	–	–
DM-BS014	10/4/2017	South	–	–	–	–	–	–	–	–
DM-BS015	10/4/2017	Triage 1	–	–	–	–	–	–	–	–
DM-BS016	10/10/2017	South	–	–	–	–	–	–	–	–
DM-BS017	10/10/2017	North	–	–	–	–	–	–	–	–
DM-BS018	10/10/2017	Triage 1	–	–	–	–	–	–	–	–
DM-BS019	10/17/2017	North	–	–	–	–	–	–	–	–
DM-BS020	10/17/2017	South	–	–	–	–	–	–	–	–
DM-BS021	10/17/2017	Triage 1	–	–	–	–	–	–	–	–
DM-BS022	10/24/2017	North	–	–	–	–	+	–	–	–
DM-BS023	10/24/2017	South	–	–	–	–	+	–	–	–
DM-BS024	10/24/2017	Triage 1	–	–	–	–	+	–	–	–
DM-BS025	10/31/2017	North	–	–	–	–	+	–	–	–
DM-BS026	10/31/2017	South	–	–	–	–	+	–	–	–
DM-BS027	10/31/2017	Triage 1	–	–	–	–	–	–	–	–
DM-BS028	11/7/2017	South	–	–	–	–	–	–	–	–
DM-BS029	11/7/2017	North	–	–	–	–	–	–	–	–
DM-BS030	11/7/2017	Triage 2	–	–	–	–	–	–	–	-
DM-BS031	11/14/2017	North	–	–	–	–	–	–	–	–
DM-BS032	11/14/2017	Triage 3	–	–	–	+	–	–	–	–
DM-BS033	11/14/2017	South	–	–	–	–	–	–	–	–
DM-BS034	11/21/2017	North	–	–	–	–	+	–	–	–
DM-BS035	11/21/2017	South	–	–	–	–	–	–	–	–
DM-BS036	11/21/2017	Triage 2	–	–	–	–	–	–	–	–
DM-BS037	11/28/2017	North	–	–	–	–	+	–	–	–
DM-BS038	11/28/2017	South	–	–	–	–	–	–	–	–
DM-BS039	11/28/2017	Triage 1	–	–	–	–	–	–	–	–
DM-BS040	1/25/2018	Triage 2	–	–	–	–	–	–	–	–
DM-BS041	1/25/2018	North	–	–	–	–	–	–	–	–
DM-BS042	1/25/2018	South	–	–	–	–	–	–	–	–
DM-BS043	2/1/2018	Triage 1	–	–	–	–	+	–	–	–
DM-BS044	2/1/2018	South	–	–	–	–	–	–	–	–
DM-BS045	2/1/2018	North	–	–	–	–	–	–	–	–
DM-BS046	2/8/2018	South	–	–	–	–	–	–	–	–
DM-BS047	2/8/2018	North	–	–	–	–	+	–	–	–
DM-BS048	2/8/2018	Triage 2	–	–	–	–	–	–	–	–
DM-BS049	2/15/2018	North	–	–	–	–	–	–	–	–
DM-BS050	2/15/2018	South	–	–	–	–	+	–	–	–
DM-BS051	2/15/2018	Triage 2	–	–	–	–	–	–	–	–
DM-BS052	2/22/2018	North	–	–	–	–	–	–	–	–
DM-BS053	2/22/2018	South	+	–	–	–	–	–	–	–
DM-BS054	2/22/2018	Triage 2	–	–	–	–	–	–	–	–
DM-BS055	3/1/2018	South	–	–	–	–	–	–	–	–
DM-BS056	3/1/2018	North	–	–	–	–	–	–	–	–
DM-BS057	3/1/2018	Triage 2	–	–	–	–	–	–	–	–

*FluA, influenza A virus; FluB, influenza B virus; FluC, influenza C virus; FluD, influenza D virus; AdV, adenovirus; CoV, coronavirus*.

**Table 2:** Typing results for positive adenovirus specimens.

**Sample**	**Human adenovirus type**	**NCBI accession number**
DM-BS022	Type 7	KU145089.1
DM-BS023	Type 1	MF085399.1
DM-BS024	Type 7	KU145089.1
DM-BS025	Type 1	MF085396.1
DM-BS037	Type 21	KM677954.1

## Discussion

In this pilot study, we conducted aerosol surveillance for human and potentially zoonotic respiratory viruses in a hospital emergency room setting. Respiratory viruses were detected in 27% of aerosol samples. Additionally, one aerosol sample had molecular evidence of influenza D virus, which is likely a rare event. Through our surveillance, we were able to detect molecular evidence of respiratory pathogens in aerosol samples through non-invasive environmental sampling techniques. Although traditional surveillance methods rely heavily on laboratory testing and clinical reports of disease activity (1), recent publications have indicated that this type of aerosol and personal sampling method may be valid as in field settings (15, 16).

Similar to Wang et al. (17), our surveillance detected the presence of adenoviruses in aerosol samples (17). As these viruses are relatively hardy DNA viruses, this finding was not unexpected as adenoviruses may circulate during respiratory virus season. Despite the use of pan-species molecular detection methods, we did not find evidence of novel or zoonotic viruses; however, as we detected influenza A and D viruses, the possibility of zoonotic viruses cannot be excluded. Despite this, given our limited number of aerosol samples, over a short period of time, it is likely that we lacked the sample size to detect airborne zoonotic viruses in a hospital setting. While we found the molecular detection of influenza D virus in a single aerosol sample very interesting, without further validation through culture or sequencing validation, or similar detections among clinically ill patients, we do not interpret the influenza D detection as worthy of public health attention.

This pilot study was limited in that we could not link aerosol results with individual patient illness or patient density seen in the ED. Although the ED did report patients with respiratory illness, and in particular influenza A and B, during the sampling period, we were not able to temporally link our findings with those patients. Additionally, although not the focus of our study, we did not detect viable viruses associated with positive aerosol samples in the ED.

Despite these limitations, the results of this study indicate that aerosol sampling is a useful complement to traditional sampling methods. Our finding that 27% of collected aerosol samples showed molecular evidence for at least one respiratory pathogen suggests that patients waiting in the emergency room are shedding virus in aerosolized droplets. As this method is non-invasive and relatively low in cost, there are advantages to environmental sampling techniques in high density areas, such as the emergency room, where direct patient sampling is difficult or not possible. Additionally, as this and other studies have demonstrated the ability to molecularly detect airborne viruses in hospital waiting room infection control procedures such as the use of N95 respirators for sick patients should be considered. This information on the molecular detection of respiratory viruses in hospital aerosols can be used to inform hospital practice on prevention and spread of infection in waiting rooms. Further study on the transmission of viruses in the air is needed to determine potential for infection with airborne particles in this type of setting.

## Author contributions

JC, JZ, SP, and MY collected and analyzed samples. JC and JZ wrote the introduction and materials and methods. EB wrote the results and discussion. GG conceived of the idea of the study and helped revise the manuscript to add important scientific content and refine the interpretation of the results. All the authors reviewed the final version of the manuscript and agreed to its submission.

## Conflict of interest statement

The authors declare that the research was conducted in the absence of any commercial or financial relationships that could be construed as a potential conflict of interest.
